# MicroRNAs Modulate Oxidative Stress in Hypertension through PARP-1 Regulation

**DOI:** 10.1155/2017/3984280

**Published:** 2017-06-04

**Authors:** Douglas F. Dluzen, Yoonseo Kim, Paul Bastian, Yongqing Zhang, Elin Lehrmann, Kevin G. Becker, Nicole Noren Hooten, Michele K. Evans

**Affiliations:** ^1^Laboratory of Epidemiology and Population Science, National Institute on Aging, National Institutes of Health, 251 Bayview Boulevard, Baltimore, MD 21224, USA; ^2^Laboratory of Genetics and Genomics, National Institute on Aging, National Institutes of Health, 251 Bayview Boulevard, Baltimore, MD 21224, USA

## Abstract

Oxidative stress is thought to contribute to aging and age-related diseases, such as cardiovascular and neurodegenerative diseases, and is a risk factor for systemic arterial hypertension. Previously, we reported differential mRNA and microRNA (miRNA) expression between African American (AA) and white women with hypertension. Here, we found that the poly-(ADP-ribose) polymerase 1 (PARP-1), a DNA damage sensor protein involved in DNA repair and other cellular processes, is upregulated in AA women with hypertension. To explore this mechanism, we identified two miRNAs, miR-103a-2-5p and miR-585-5p, that are differentially expressed with hypertension and were predicted to target *PARP1*. Through overexpression of each miRNA-downregulated PARP-1 mRNA and protein levels and using heterologous luciferase reporter assays, we demonstrate that miR-103a-2-5p and miR-585-5p regulate *PARP1* through binding within the coding region. Given the important role of PARP-1 in DNA repair, we assessed whether overexpression of miR-103a-2-5p or miR-585-5p affected DNA damage and cell survival. Overexpression of these miRNAs enhanced DNA damage and decreased both cell survival and colony formation. These findings highlight the role for PARP-1 in regulating oxidative DNA damage in hypertension and identify important new miRNA regulators of PARP-1 expression. These insights may provide additional avenues to understand hypertension health disparities.

## 1. Introduction

Systemic arterial hypertension, an age-associated chronic disease, is a predictor of vascular-associated mortality from stroke and ischemic heart disease [1, 2]. It affects more than 80 million people ≥ 20 years old in the United States with increased prevalence over the lifespan [2]. Hypertension disproportionally affects African Americans (AAs) in the United States, particularly in AA women with a prevalence of >46%, and AAs develop hypertension earlier than whites or any other ethnicity in the United States [2]. Even at similar levels of systolic blood pressure, AAs have a higher risk of stroke compared with whites and death rates attributed to hypertension are higher in both male and female AAs compared with whites or Hispanics [2, 3].

The genetic and molecular factors contributing to hypertension disparities are still largely unknown. Perturbations in the renin-angiotensin-aldosterone system (RAAS) and nitric oxide (NO) pathway in immune cells and in the endothelium can contribute to the development of hypertension [4, 5], but how these pathways influence disparities in hypertension etiology is not clear. Healthy AA men have elevated production of superoxides in peripheral blood mononuclear cells (PBMCs) compared with white men [6], but investigation of these pathways in healthy or hypertensive AA women has not been investigated. Previously, we identified significant differential gene expression profiles by race and disease status in PBMCs isolated from AA and white women with or without hypertension [7]. We observed that gene expression in hypertension-related pathways, in RAAS and other inflammatory pathways, was significantly upregulated in AA hypertensives compared with both normotensive controls and white hypertensives [7].

Gene expression can be regulated by microRNAs (miRNAs), short, single-stranded RNAs which posttranscriptionally regulate protein expression by binding to target mRNAs and inhibiting translation and promoting transcript degradation [8]. miRNAs also can affect transcription by regulating various transcriptional pathways including members of the SWI/SNF chromatin remodeling complex [9]. We examined miRNA expression in AA and white normotensive and hypertensive females and identified differentially expressed miRNAs that regulate hypertension-related targets in endothelial cells [7]. MiRNA regulation is essential for proper immune cell [10] and endothelial function [11, 12], and altered regulation and expression of these genes can contribute to endothelial dysfunction [13]. miRNA profiles can also serve as biomarkers for cardiovascular diseases (CVDs), such as peripheral arterial disease (PAD) [14].

Differential gene expression in both mRNAs and miRNAs may play a role in hypertension disparities through modulating oxidative stress. Meta-analysis of gene expression datasets of blood samples from patients with cardiovascular disease (CVD) identified differentially expressed genes in oxidative stress and related inflammatory pathways [15]. Endothelial cells from AA women with PAD have increased oxidative stress and reactive oxygen species (ROS) compared to AA men and white women, but the gene expression differences contributing to this were not investigated [16]. Oxidative stress can cause hypertension by promoting ROS production, including the overexpression of nicotinamide adenine nucleotide phosphate (NADPH) oxidases and cyclooxygenases (COXs), which can reduce NO signaling in the endothelium and cause endothelial dysfunction [17–20]. Higher levels of ROS production and oxidative stress can also lead to DNA damage, further promoting inflammatory pathways contributing to hypertension etiology.

Specialized DNA repair pathways are activated by DNA damage leading to signaling cascades that resolve the base or strand damage. Poly-(ADP-ribose) polymerase 1 (PARP-1) is a DNA damage sensor protein that initiates and propagates DNA repair through poly-ADP-ribosylation of key DNA repair proteins [21, 22]. Many other cellular processes also rely on PARP-1 signaling, and abnormal PARP-1 activity has been attributed to various age-related diseases, including hypertension [23–25]. Consistent with these findings, inhibiting PARP-1 activity in endothelial cells protects against endothelial dysfunction [26, 27] and PARP-1 inhibitors are under consideration as a treatment for pulmonary hypertension [28]. However, differential expression of PARP-1 has not been investigated with respect to health disparities in essential hypertension. Furthermore, racial differences in oxidative stress gene pathways in hypertension have not been fully explored.

We report here that AA women with hypertension exhibit elevated expression of genes in oxidative stress and DNA damage response pathways. Through bioinformatic and pathway analysis, we have identified two novel miRNA regulators of PARP-1 protein expression, miR-103a-2-5p and miR-585-5p, that influence the DNA damage response and cell survival in primary endothelial cells. Identifying the mechanisms governing differential expression of oxidative stress and inflammatory pathways could ultimately provide novel avenues to explore for therapeutic intervention.

## 2. Materials and Methods

### 2.1. Study Participants

A subcohort of age-matched, African American (AA) and white females who were either normotensive (NT) or hypertensive (HT) were chosen from the Healthy Aging in Neighborhoods of Diversity across the Life Span (HANDLS) study of the National Institute on Aging Intramural Research Program (NIA IRP), National Institutes of Health (NIH). More information about the HANDLS cohort can be found in [29]. The Institutional Review Board of the National Institute of Environmental Health Sciences, NIH, approved this study, and all participants signed written informed consent documents. The subcohort consists of WNT, WHT, AANT, and AAHT (*n* = 20/group for validation) females, and more extensive demographic and clinical information about this cohort have been previously described [7].

### 2.2. Cell Culture, Transfection, and RNA Isolation

Primary human aortic endothelial cells (HAEC) were grown in EMB-2 supplemented with the EGM-2 SingleQuot Kit (Lonza; Walkersville, MD). HUVECs were grown in EBM supplemented with the EGM SingleQuot Kit (Lonza). HeLa cells were grown in Dulbecco's modified Eagle's medium (DMEM, Invitrogen) and supplemented with 10% fetal bovine serum (FBS). Cells were transfected with pre-miR miRNA precursors for hsa-miR-103a-2-5p, hsa-miR-585-5p, or scrambled (Scr) negative control (Life Technologies) using Lipofectamine 2000 (Life Technologies). Total RNA from HUVECs and HAECs was isolated using TRIzol (Life Technologies). RNA quality was measured by a Nanodrop 2000.

### 2.3. Microarray Analysis and Target Prediction

**Figure 1:**
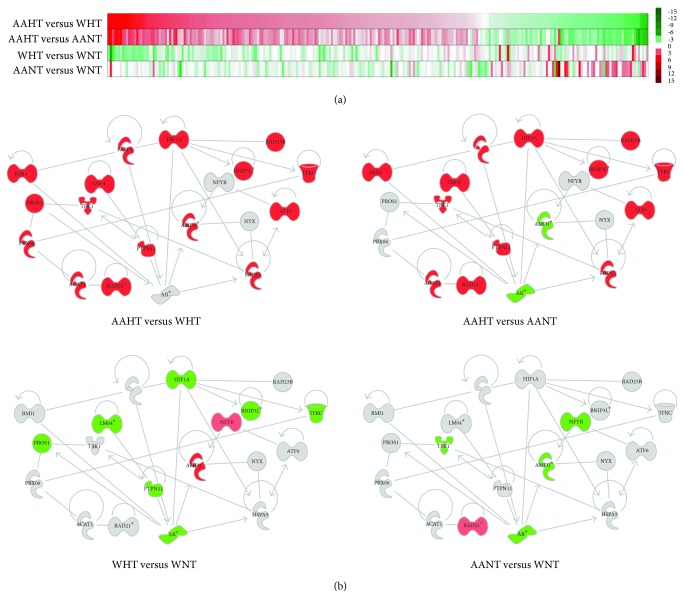
Pathway analysis of gene expression in hypertensive women. (a) Heat map of significantly changed genes identified from top canonical and physiological pathways and functions in HAECs after overexpression of miR-103a-20-5p and miR-585-5p miRNA mimics. Red (up) and green (down) indicate relative Z-ratios of significantly and-differentially expressed mRNAs in PBMCs from our hypertension cohort and reflect changes in AAHT compared with WHT or AANT, WHT with WNT, and AANT with WNT. (b) IPA-curated, hierarchal clustering of functional relationships between individual genes predicted to be targets of either miR-103a-2-5 or miR-585-5p. Red (up) and green (down) indicate relative fold change of significantly and differentially expressed mRNAs in PBMCs from our hypertension cohort in each comparison. AAHT: AA hypertensives; AANT: AA normotensives; WHT: white hypertensives; WNT: white normotensives.

mRNA expression in HAECs was analyzed by microarray using the Illumina Beadchip HT-12v4 (Illumina, San Diego, CA). RNA was prepared and labeled per the manufacturer's protocol. Raw signal data were subjected to Z-score normalization per [30] to ensure compatibility and normalization. Individual genes with Z-ratios < −1.5-fold, *p*  value < 0.05, and average intensity > 0 were considered significant. The DIANA-microT prediction algorithm [31] and Ingenuity Pathway Analysis (IPA; Ingenuity Systems, Redwood City, CA) were used to identify mRNA targets for miR-103a-2-5p and miR-585-5p as well as to visualize functional, hierarchical relationships between target predictions (as seen in Figure 1()). IPA network analysis was performed as previously described [7], using both the default setting analysis and custom prediction modeling features. All microarray data can be found on Gene Expression Omnibus GSE95431.

### 2.4. RT-qPCR Analysis

mRNA from HAECs and HUVECs was transcribed into cDNA using random hexamers and Script II reverse transcriptase (Invitrogen), and miRNA was transcribed into cDNA using the QuantiMiR RT Kit (Systems Biosciences, Mountain View, CA). Transcript levels were assessed by RT-qPCR using 2x SYBR green master mix (Applied Biosystems) on an Applied Biosystems model 7500 real-time PCR machine. *PARP1* levels were normalized to the average of *ACTB* and *GAPDH*, and miR-103a-2-5p and miR-585-5p were normalized to *U6.* All gene expression levels were calculated using the 2^-ΔΔCt^ method [32]. The following primers were used (forward and reverse, respectively, for each mRNA): GGACTTCGAGCAAGAGATGG and AGCACTGTGTTGGCGTACAG for *ACTB,* GCTCCTCCTGTTCGACAGTCA and ACCTTCCCCATGGTGTCTGA for *GAPDH,* and CGAGATCATCAGGAAGTATGTTAAGAA and GCTGGCATTCGCCTTCAC for *PARP1*. The following forward primers were used for each miRNA and used with the U6 forward primer and universal reverse primer provided with the QuantiMiR RT Kit: AGCTTCTTTACAGTGCTGCCTTG for miR-103a-2-5p and CTAGCACACAGATACGCCCAGA for miR-585-5p.

### 2.5. Western Blot Analysis

HAECs and HUVECs were washed twice with cold PBS and lysed in 2x Laemmli sample buffer. Lysates were loaded onto a 10% acrylamide gel, and protein levels were assessed by immunoblotting with anti-PARP-1 (Cell Signaling) and anti-GAPDH (Santa Cruz, Dallas, TX) or anti-Actin (Santa Cruz) antibodies.

### 2.6. Luciferase Reporter Assays

The cDNA fragments corresponding to the partial *PARP1* mRNAs were amplified by PCR using specific primers. After XhoI and NotI digestion, the PCR product was cloned downstream of the Renilla open reading frame of the psiCHECK2 reporter plasmid. HeLa cells were transfected with 500 ng of the indicated luciferase reporter constructs and transfected again 24 hrs later with the miRNA mimics or Scr control. Twenty-four hours later, RL and FL activities were measured using the Dual-Luciferase® Reporter Assay System (Promega) per the manufacturer's instructions. The following primers were used (forward and reverse, respectively): GCATCTCGAGATGGCGGAGTCTTCGGATAAGC and GCATGCGGCCGCTGTGGAGGGC GGAGGCGTG for *PARP1(CR-a)* and GCATCTCGAGGGTACGGTGATCGGTAGCAA and GCATGCGGCCGCCTTGTAACGCTGGCATTCGC for *PARP1(CR-b).*

### 2.7. Single Cell Gel Electrophoresis (Comet) Assays

Forty-eight hours after transfection with pre-miRNA precursors for hsa-miR-103a, hsa-miR-585-5p, or Scr control, HAECs were untreated or treated with 100 *μ*M H_2_O_2_ for 30 min in serum-free EMB-2. This concentration of H_2_O_2_ induces various base lesions and DNA breaks. Single-cell alkaline comet assays were performed essentially as described [33, 34]. Comets were imaging on an Eclipse E-400 fluorescence microscope (Nikon, Japan) attached to a Pulnix video camera (Kinetic Imaging, LTD, Liverpool, UK) and were quantified using Komet 5.5 software (Kinetic Imaging LTD). Olive tail moment was used as a measure of DNA damage level [35, 36].

### 2.8. Immunofluorescence

Forty-eight hours after transfection, HAECs were untreated or treated with 10 *μ*M menadione for 30 min in serum-free EMB-2. Cells were stained for 8-oxo-7,8-dihydroguanine (8-oxoG) and 4′,6-diamidino-2-phenylindole (DAPI) as previously described [37] using an anti-8-oxoG monoclonal antibody from Millipore and Alexa-568 conjugated secondary antibodies from Invitrogen. Images were taken on a Zeiss Observer D1 microscope with an AxioCam1Cc1 camera at a set exposure, and fluorescence intensity was measured for each 8-oxoG stained nuclei using AxioVision Rel 4.7 software.

### 2.9. Colony Formation and Cell Survival Assays

For the colony formation assays, HAECs were transfected with pre-miRNA precursors miR-103a-2-5p or miR-585-5p cells or Scr control, and 24 hrs later, cells (3000/well) were plated in 6-well plates in triplicate and allowed to attach for 24 hrs. HAECs were treated with or without 50 *μ*M H_2_O_2_ for 7 days. The media with or without 50 *μ*M H_2_O_2_ was changed once during the time of the assay. Colonies were stained with 0.5% Crystal violet in 50% methanol. Plates were washed with PBS and were imaged. Only colonies with >100 cells were counted.

Scr control, miR-103a-2-5p, or miR-585-5p cells (5000/well) were plated in a 96-well plate. The following day, cells were untreated or treated for 24 h with 100 *μ*M H_2_O_2_. Cell survival was measured using a MTT assay (Sigma).

### 2.10. Statistical Analysis

Student's *t-*test was used when comparing two groups, unless otherwise noted. A *p* value of <0.05 was considered statistically significant.

## 3. Results

### 3.1. Identification and Analysis of Hypertension-Related MicroRNAs and Genes

We previously reported that there is significant, differential mRNA and miRNA expression by race and/or hypertension status in PBMCs isolated from AA and white women. Nine miRNAs were differentially expressed in our cohort. We bioinformatically identified and confirmed novel hypertension-related mRNA targets for five of these miRNAs in primary human endothelial cells [7]. In the present study, we sought to identify and confirm novel hypertension-related mRNA targets for two more miRNAs differentially expressed in our previous cohort, miR-103a-2-5p and miR-585-5p, which until then had never been associated with essential hypertension.

We have identified potential mRNA targets for miR-103a-2-5p and miR-585-5p using the DIANA-microT prediction algorithm [31] and Ingenuity Pathway Analysis (IPA). miR-103a-2-5p and miR-585-5p are predicted to target 4031 and 255 mRNAs, respectively (Supplemental Dataset 1 available online at https://doi.org/10.1155/2017/3984280). To confirm these predictions in vitro, we individually overexpressed miR-103a-2-5p, miR-585-5p, and scrambled (Scr) control miRNA mimics in primary human aortic endothelial cells (HAECs). Total RNA was isolated to assess global gene expression levels via microarray. A total of 1178 and 1112 unique mRNAs were significantly downregulated >1.5-fold in the presence of miR-103a-2-5p and miR-585-5p mimics, respectively (Supplemental Dataset 1). For miR-103a-2-5p, twenty-nine mRNAs were both repressed in HAECs and differentially expressed in hypertension-related pathways in PMBCs in our previous cohort; however, there were no targets identified for miR-585-5p using this approach (Supplemental Data Set 1).

We next used IPA to identify the top canonical and physiological pathways and functions in HAECs that exhibited significant gene expression differences in the presence of each miRNA mimic (Table 1; Columns II and III). Additionally, we identified which genes from each pathway that were significantly repressed by each miRNA mimic and differentially expressed in PBMCs in our hypertension cohort (Table 1). Of the top five, significantly altered canonical pathways, miR-103a-2-5p was predicted to regulate *HSPA5* and *RAD21*, while there were no targets identified for miR-585-5p. Of the top significantly changed molecular, cellular, and physiological pathways in HAECs, miR-103a-2-5p and miR-585-5p were predicted to target mRNAs related to DNA replication, recombination, and repair and cellular growth and proliferation. miR-103a-2-5p and miR-585-5p were both predicted to target *PARP1*, and miR-103a-2-5p was predicted to regulate the expression of fourteen additional mRNAs related to these pathways (Table 1).

We next sought to identify any disease or racial differences in gene expression of these pathways in our hypertension cohort. We generated a heat map of the Z-ratios of all genes in PBMCs from our hypertension cohort that are found in the pathways significantly affected by miR-103a-2-5p and miR-585-5p overexpression in HAECs (Figure 1(a)). We observed that AA women with hypertension (AAHT) had significantly elevated global gene expression in these pathways with respect to both AA normotensive controls (AANT) and white women with hypertension (WHT). Gene expression levels were decreased in WHT compared with white normotensive controls (WNT), and in general, few genes were significantly different when comparing AANT with WNT. Of those that were significantly different, they were most often upregulated in AANT (Figure 1(a)). These data suggest that there are unique genes and gene expression profiles differentially expressed and associated with hypertension status and race that are potentially regulated by miR-103a-2-5p and miR-585-5p.

We next used IPA to build a curated, hierarchal cluster of functional relationships between individual genes predicted to be targets of each miRNA, significantly repressed in HAECs and differentially expressed in our hypertension cohort (Figure 1(b)). We observed that *PARP1* and related genes are significantly overexpressed in AAHT compared with WHT or AANT, reflecting the global profiles discussed above. The same genes are predominately repressed in WHT compared to WNT, and most genes, except for *RAD21*, *TBK1*, *NFYB*, *AMD1*, and *AR,* are not differentially expressed when comparing AA and white normotensive controls (Figure 1(b)). These data suggest that genes involved with DNA repair, endothelial cell growth, and cardiovascular function exhibit differential expression in hypertension by race and may contribute to differences observed in hypertension health disparities in women [7].

### 3.2. miR-103a-2-5p and miR-585-5p Regulate PARP1 Expression

**Figure 2:**
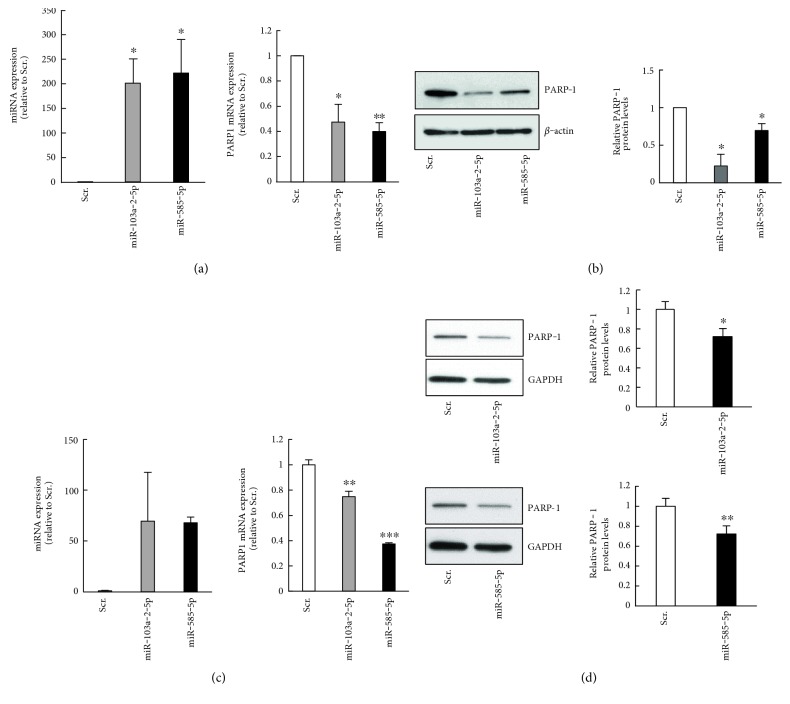
miR-103-2-5p and miR-585-5p decrease PARP-1 expression. HAECs (a, b) or HUVECs (c, d) were transfected with precursor mimics for miR-103-2-5p, miR-585-5p, or scrambled (Scr) control. After 48 hrs, cells were lysed for RNA and protein analysis. (a, c) miRNA and *PARP1* mRNA levels were quantified by RT-qPCR analysis. (b, d) Lysates were analyzed by SDS-PAGE and immunoblotted with anti-PARP-1, anti-GAPDH, and anti-*β*-actin antibodies. Histograms represent the mean + SEM and from three independent experiments ^∗^*P* < 0.05 and *P* < 0.01 by Student's *t*-test.

Given that miR-103a-2-5p and miR-585-5p are both predicted to target *PARP1*, and PARP-1 has previously been implicated in hypertension pathology, we examined whether miR-103a-2-5p and miR-585-5p regulate PARP-1 expression. To test this, we overexpressed these miRNAs by transfecting precursors into human aortic endothelial cells (HAEC) and human umbilical vein endothelial cells (HUVEC). miR-103a-2-5p and miR-585-5p overexpression decreased PARP-1 mRNA and protein abundance (Figure 2), indicating that PARP-1 levels can be modulated by these miRNAs.

**Figure 3:**
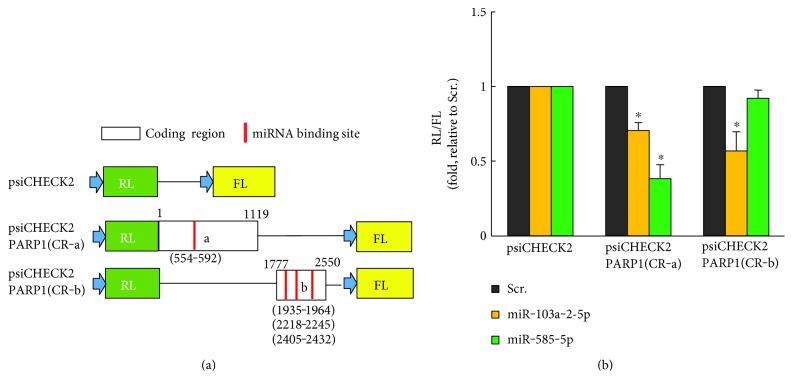
miR-103-2-5p and miR-585-5p target PARP-1. (a) Schematic of *PARP1* coding region (CR) dual-luciferase reporter constructs. psiCHECK2 control plasmid expresses both the Renilla luciferase (RL) and Firefly luciferase (FL). Constructs of the *PARP1(CR)* span the indicated predicted miRNA binding sites (red color bars). (b) Cells were transfected with the dual-luciferase constructs and the indicated miRNAs or scrambled control (Scr). The ratio of RL/FL activity is shown. The histogram represents the mean + SEM from three independent experiments. ^∗^*P* < 0.05 by Student's *t*-test compared to Scr.

To further confirm that miR-103a-2-5p and miR-585-5p regulate PARP-1 expression, we used heterologous luciferase reporter plasmids (Figure 3) that express Renilla luciferase (RL) from constructs lacking or containing the *PARP1 CR* (psiCHECK2 or psiCHECK2-*PARP1-CR-a* and *CR-b*). These plasmids also contain Firefly luciferase (FL), which served as an internal transfection control. The ratio of RL/FL activity from each transfected reporter plasmid indicated that miR-103a-2-5p significantly decreased the levels of psiCHECK2-*PARP1 CR*-*a* and *CR-b*. miR-585-5p reduced the levels of psiCHECK2-*PARP1 CR-a,* while it did not affect the activity of the psiCHECK2- *PARP1 CR-b* reporter (Figure 3(b)). These data suggest that miR-103a-2-5p and miR-585-5p regulate PARP-1 through areas in the *PARP1* coding region. Although uncommon, miRNA regulation of mRNA transcripts in the coding region can inhibit translation [38]. Bioinformatic analyses using DIANA-microT [31] predicted that miR-103a-2-5p has four binding sites within the *PARP1* coding region (CR) but did not identify a binding site for miR-585-5p. We observed that miR-585-5p does functionally regulate the *PARP1* coding region and this may be through a noncanonical miRNA binding site [39, 40]. We identified potential noncanonical sites for miR-585-5p in this area of the *PARP1* coding region. Target prediction analysis did not identify binding sites for either miRNA in the 3′UTR of *PARP1*.

### 3.3. miR-103a-2-5p and miR-585-5p Enhance DNA Damage

**Figure 4:**
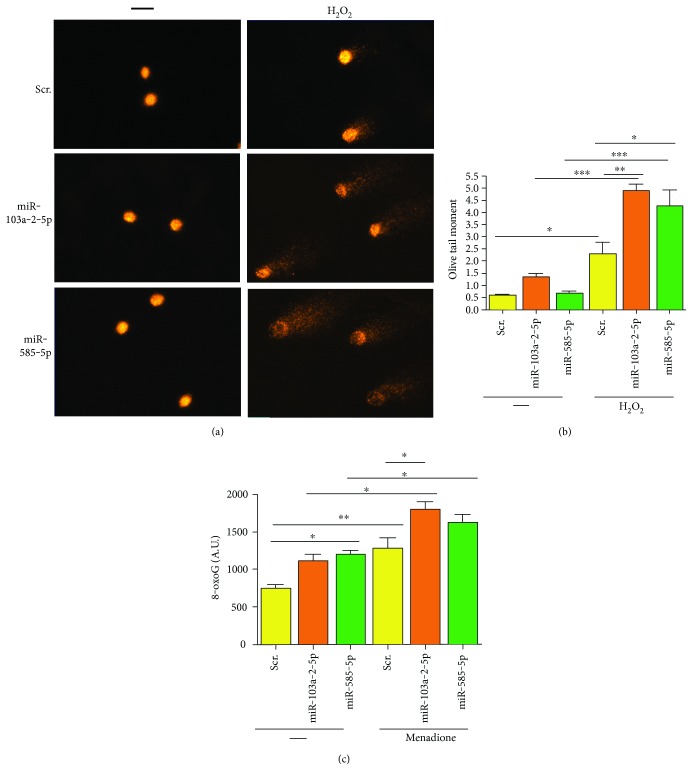
miR-103a-2-5p and miR-585-5p enhance DNA damage. HAECs were transfected with precursor mimics of miR-103a-2-5p, miR-585-5p, or scrambled control (Scr). Forty-eight hrs after transfection, cells were untreated (−) or treated with 100 *μ*M H_2_O_2_ for 30 min and comet assays were performed under alkaline conditions. Representative comets are shown in (a), and olive tail moment was used as a measure of DNA damage (b). (c) Forty-eight hours after transfection with the indicated miRNAs, HAECs were untreated (−) or treated with 10 *μ*M menadione (men) for 30 min. Cells were stained with anti-8oxoG antibodies, and fluorescent intensity was calculated as described in the Materials and Methods. The histograms represent the mean of 3 independent experiments + SEM. ^∗^*P* < 0.05, ^∗∗^*P* < 0.01, ^∗∗∗^*P* < 0.001 for the indicated comparisons using one-way ANOVA and Tukey's post hoc test.

Given the important role that PARP-1 plays as a DNA damage sensor and in DNA repair, we assessed whether overexpression of miR-103a-2-5p and miR-585-5p enhanced DNA damage. To initially test this idea, we examined whether miR-103a-2-5p and miR-585-5p affected DNA damage levels using the single-cell gel electrophoresis (comet) assay under alkaline conditions, which measures alkaline-sensitive sites including DNA breaks, alkaline labile sites, and transient repair sites. In the absence of DNA damage, there was very little difference in the amount of endogenous DNA damage between scrambled control and miR-585-5p and miR-103a-2-5p transfected cells, although there was a slight enhancement of DNA damage in miR-103a-2-5p transfected cells, but this did not reach significance (Figures 4(a) and 4(b)). However, after treatment with the DNA-damaging agent H_2_O_2_, there was a significant increase in the amount of DNA damage in both miR-585-5p and miR-103a-2-5p transfected cells compared with control transfected cells (Figures 4(a) and 4(b)).

We also addressed whether miRNA overexpression affected levels of the DNA base lesion, 8-oxoG, which can be detected in HAECs by immunostaining. DNA base lesions, including 8-oxoG, are repaired by proteins in the base excision repair pathway, including PARP-1. Endogenous 8-oxoG levels were higher in cells transfected with miR-103a-5-5p or miR-585-5p (Figure 4(c)). Treatment with the DNA-damaging agent menadione induces DNA base lesions. miR-103a-2-5p or miR-585-5p transfection increased 8-oxoG levels over control transfected cells after treatment with menadione. In summary, these data indicate that miR-103a-2-5p and miR-585-5p enhance DNA damage.

### 3.4. miR-103a-2-5p and miR-585-5p Decrease Cell Survival and Colony Formation

**Figure 5:**
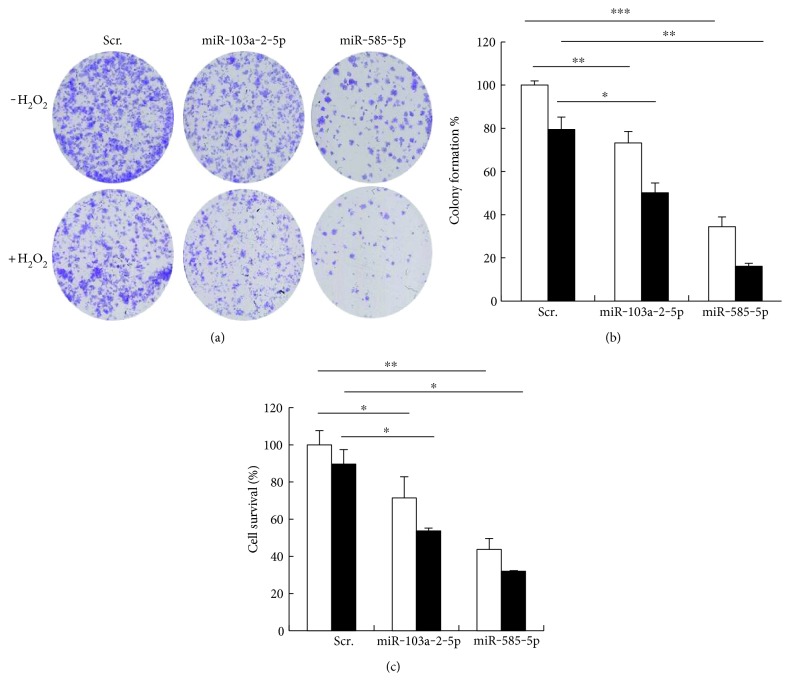
miR-103-2-5p and miR-585-5p decrease colony formation and cell survival. (a, b) HAECs transfected with miR-103a-2-5p, miR-585-5p, or scrambled control (Scr.) were plated, and 24 hrs later, cells were either untreated or treated with 50 *μ*M H_2_O_2_. After 7 days, colony formation was imaged and quantified. (c) HAECs were treated with 100 *μ*M H_2_O_2_ for 24 hrs, and cell survival was measured by the MTT assay. The histograms are normalized to Scr untreated and represent the mean + SEM from three independent experiments. ^∗^*P* < 0.05, ^∗∗^*P* < 0.01, ^∗∗∗^*P* < 0.001 by Student's *t*-test.

Given that higher levels of DNA damage can sometimes decrease cell survival, we investigated whether miR-103a-2-5p and miR-585-5p affected cell survival with both short or prolonged exposures to DNA-damaging agent*s.* miR-103a-2-5p and miR-585-5p transfected cells were exposed to a prolonged low dose of H_2_O_2_ to mimic physiological treatment conditions. Overexpression of these miRNAs significantly decreased colony formation with or without H_2_O_2_ treatment (Figures 5(a) and 5(b)). We also treated cells with a higher dose of H_2_O_2_ for a shorter time to address the effects on cell survival. Similar to the colony formation assays, overexpression of miR-103a-2-5p and miR-585-5p significantly reduced cell survival both in the presence and absence of DNA damage (Figure 5(c)). Collectively, these data suggest that miR-103a-2-5p and miR-585-5p reduce endothelial cell survival potentially through modulation of PARP-1 expression.

## 4. Discussion

Previously, we identified miRNAs that were differentially expressed by race and/or hypertension in AA and white women [7]. Our data suggested that differential expression of various miRNA : mRNA pairs may contribute to hypertension etiology. Here, we focused on two miRNAs we identified in our previous studies to better understand mechanistically how they may contribute to hypertension. We found, using bioinformatic analysis based on our gene and miRNA expression levels in hypertensives and subsequent target validation, that miR-103a-2-5p and miR-585-5p both target the DNA damage sensor and repair protein PARP-1. In the context of racial differences in hypertension, we observed higher levels of *PARP1* in AAHT compared to AANT. *PARP1* levels were also higher in AAHT compared to WHT, but not WHT compared to WNT. These data suggest that differential *PARP1* expression is important in hypertension and may have a racial component.

In addition to the miRNAs we found to target *PARP1*, other miRNAs have also been shown to target *PARP1*, including miR-335, miR-520, miR-489, and miR-223 [41–44]. miR-223 is downregulated in the lungs, arteries, and smooth muscle cells of patients with pulmonary hypertension. This decrease in miR-223 expression was regulated by increased activity of hypoxia-inducible factor 1*α* (HIF1*α*), and this in turn upregulated PARP-1 activity and contributed to endothelial dysfunction. Restoration of miR-223 levels decreased PARP-1 activity and restored endothelial function [43]. Therefore, we cannot exclude that these other miRNAs, such as miR-223, may play a role in targeting PARP-1 expression in hypertension. However, we did not observe any significant changes in these miRNAs with race or hypertension status in our previous study [7], which indicates that in this context, miR-103a-2-5p and miR-585-5p have roles in systemic arterial hypertension physiology in PARP-1 regulation.

Ample evidence has shown that oxidative stress contributes to hypertension through excessive production of both oxygen and nitrogen-derived free radicals [20, 45]. These species in turn cause DNA damage which can activate PARP-1. PARP-1 plays an important role in repairing damage, generally through poly-ADP-ribosylation of key DNA repair proteins. On the contrary, excessive PARP-1 activation can have detrimental cellular effects through depletion of NAD+ stores and increasing inflammatory pathways [25]. An increase in inflammation can further contribute to hypertension through a myriad of effects including the recruitment and accumulation of inflammatory cells to vessels [20].

As a model system to test the functional interaction of miR-103a-2-5p and miR-585-5p with PARP-1, we overexpressed these miRNAs in primary aortic and umbilical vein endothelial cells. Our overexpression studies, combined with our luciferase reporter assays, indicate that these miRNAs can target *PARP1*. Furthermore, we found that miR-103a-2-5p and miR-585-5p enhanced oxidant-induced DNA damage and cell death. These data are consistent with previous reports that inhibiting PARP-1 enhances DNA damage and decreases cell survival [37, 46]. In contrast, PARP-1 has also been shown to promote endothelial cell survival in response to oxidative and nitrosative stress [47]. Therefore, PARP-1 plays a complex dual role. The complexity of these roles is illustrated by the fact that mice lacking PARP-1 or overexpressing PARP-1 die prematurely due to age-related pathologies [25, 48]. In addition, PARP-1 activity and binding to DNA repair proteins are also altered with age [25, 49–51] which may also be relevant considering hypertension increases with age. PARP-1 regulates other cellular processes including transcription, chromatin remodeling, and cell cycle regulation, which may also explain these findings [25]. Nevertheless, these data all point to the important role of PARP-1 in regulating cellular homeostasis.

We used gene expression data from PBMCs to identify and validate miRNA : mRNA regulatory pairs that may play a role in the etiology of hypertension. Previously, we found that miR-585-5p in AAHT was significantly downregulated compared with AANT and WHT [7]. Here, we found that *PARP1* expression in PMBCs is increased in AAHT compared with AANT and WHT. Although we validated this functional interaction in endothelial cells, to what degree miR-585-5p regulates *PARP1* expression in PBMCs and how that contributes to hypertension etiology remains to be elucidated. miR-103a-2-5p was also predicted to target *HIF1A*, which regulates the expression of other miRNAs that can regulate *PARP1* expression [43]. We observed that *HIF1A* expression is elevated in AAHT compared with AANT and WHT, and it is possible that miR-103a-2-5p is regulating both PARP1 and indirect upstream regulators, such as HIF1*α*. The complete pathway of PARP1 regulation will need to be further examined to understand its role in hypertension disparities.

## 5. Conclusions

Here, we have identified that PARP1 is differentially expressed with hypertension and race. Furthermore, we identified that the hypertension-related miRNAs in PBMCs, miR-103a-2-5p and miR-585-5p, target *PARP1* in endothelial cells. Our data identifies genomic factors that appear to differentially influence the presence of hypertension among African American and white women. These findings may be useful in developing personalized medicine approaches to the treatment of hypertension in populations at disproportionate risk.

## Supplementary Material

Supplementary Dataset 1: Predicted MicroRNA Target List.

## Figures and Tables

**Table 1:** Summary of HAEC pathway analysis.

MicroRNA	II. Top canonical pathways in HAECs	*P* value	DIANA or IPA-predicted pathway targets repressed ≥ 1.5-fold in HAECs & differentially expressed in PBMCs	III. Top molecular and cellular functions and physiological systems in HAECs	*P* value	DIANA or IPA-predicted pathway targets repressed ≥ 1.5-fold in HAECs & differentially expressed in PBMCs
miR-103a-2-5p	EIF2 signaling	1.4 × 10^−10^	N/A	Cell death and survival	1.6 × 10^−3^–4.3 × 10^–22^	ATF6, BMI1, BNIP3L, HIF1A, HSPA5, LMO4, **PARP1**, PTPN11, RAD21, RAD23B, TFRC
	Protein ubiquitination	3.2 × 10^–8^	HSPA5	Cell cycle	1.8 × 10^−3^–1.1 × 10^–20^	BMI1, HIF1A, RAD21, TFRC
	Interferon signaling	4.5 × 10^−8^	N/A	Cellular assembly and organization	1.5 × 10^−3^–1.1 × 10^−20^	**PARP1**, RAD21
	Mitotic roles of polo-like kinase	7.9 × 10^−8^	RAD21	DNA replication, recombination, and repair	1.6 × 10^−3^–1.1 × 10^−20^	BMI1, **PARP1**, RAD23B, PARP1
	Cell cycle control of chromosomal replication	1.6 × 10^–7^	N/A	Cellular growth and proliferation	1.8 × 10^−3^–9.1 × 10^−19^	ACAT1, AMD1, BMI1, HIF1A, HSPA5, **PARP1**, PROS1, RAD21, TFRC, TPN11
				Cardiovascular system development and function	1.3 × 10^−3^–4.1 × 10^–7^	HSPA5, PROS1
miR-585-5p	EIF2 signaling	2.3 × 10^–15^	N/A	Cell death and survival	6.9 × 10^−4^–2.1 × 10^–22^	**PARP1**
	Mitotic roles of polo-like kinase	6.6 × 10^–9^	N/A	Cell cycle	7.7 × 10^−4^–5.2 × 10^–21^	N/A
	Cell cycle control of chromosomal replication	9.2 × 10^–9^	N/A	Cellular assembly and organization	7.6 × 10^−4^–1.1 × 10^−19^	**PARP1**
	Regulation of eIF4 and p70S6K signaling	3.3 × 10^–8^	N/A	DNA replication, recombination, and repair	5.4 × 10^−4^–1.1 × 10^−19^	**PARP1**
	mTOR signaling	7.5 × 10^–8^	N/A	Cellular growth and proliferation	5.3 × 10^−4^–9.1 × 10^−17^	N/A
				Cardiovascular system development and function	6.8 × 10^−4^–1.2 × 10^–10^	**PARP1**
